# Management of radial clubhand with gradual distraction followed by centralization

**DOI:** 10.4103/0019-5413.53461

**Published:** 2009

**Authors:** Narender Saini, Purnima Patni, SP Gupta, Lokesh Chaudhary, Vishwadeep Sharma

**Affiliations:** Department of Orthopaedics, SMS Medical College and Attached Hospitals, Jaipur, India

**Keywords:** Centralization, radial clubhand, radial ray defects, serial casting, JESS

## Abstract

**Background:**

Treatment of radial clubhand has progressed over the years from no treatment to aggressive surgical correction. Various surgical methods of correction have been described; Centralization of the carpus over the distal end of the ulna has become the method of choice. Corrective casting prior to centralization is an easy and effective method of obtaining soft tissue stretching before any definitive procedure is undertaken. Moreover, it helps put the limb in a correct position. The outcome of deformity correction by serial casting / JESS distractor followed by centralization is discussed.

**Materials and Methods:**

In a prospective study, of 17 cases with 18 radial clubhands of Heikel's Grade III and IV (with average age 11 months (range 20 days – 24 months) with M:F of 2.6:1, were treated by gradual soft tissue stretching using corrective cast (14 cases) and JESS distraction (4 cases), followed by centralization (16 cases) or radialization (2 cases) and tendon transfers.

**Results:**

The average correction attained during the study was 71° of radial deviation and 31° of volar flexion. The average third metacarpal to distal ulna angle in anteroposterior and lateral view at final follow-up was 7° in both views. Angle of movement at elbow showed a small increase from 99° to 101° during the follow-up period. However, the range of movement at fingers showed increase in stiffness during the follow-up. No injury occurred to the distal ulnar epiphysis during the operative intervention. The results at the final follow-up, at the end of 2 years were graded on the basis of the criteria of F.W. Bora, and of Bayne and Klug. Considering the criteria of F.W. Bora, satisfactory result was shown by nine of the 18 hands (50%) while 16 out of 18 hands (89%) showed good or satisfactory result based on deformity criteria of Bayne and Klug.

**Conclusion:**

The management of radial clubhand by gradual corrective cast or JESS distractor followed by centralization and tendon transfers in children is an acceptable method of treatment with consistently satisfactory results, both functional and cosmetic.

## INTRODUCTION

Congenital radial clubhand is a longitudinal, radial developmental arrest characterized by varying degree of deficiencies of the radius, carpal bones, and the thumb that produces radial deviation of the hand and marked shortening of the forearm. The radial or prexial deficiencies have been classified by Heikle^1^ into four types as shown in Table 1.

**Table 1:** Heikel's classification of radial clubhand

Type I	Short distal radius
Type II	Hypoplastic radius
Type III	Partial absence of radius
Type IV	Complete absence of radius

Treatment of radial clubhand has progressed over the years from no treatment to aggressive surgical correction. In an untreated radial clubhand, although the deformity did not change much, the hand's prehensile function was never able to develop. Various surgical methods of correction have been described, such as soft tissue releases with or without ulnar osteotomy,^2^ proximal fibular transplants,^3^ arthodesis,^4^ centralization,^5^,^6^ correction by distraction in an external fixator,^7^ radialization,^8^ and Ilizarov's methods.^9^ Centralization of the hand over the distal ulna is still the basis for the modern-day treatment of this condition.^2^

Corrective casting prior to centralization is an easy and effective method of obtaining soft tissue stretching before any definitive procedure is undertaken. Moreover, it helps put the limb in a correct position from the beginning so that the child's prehensile functions are developed with a hand in corrected position. Casting also prevents extensive soft tissue releases, which were required prior to centralization in conventional procedures.^10^–^13^ This study conducted at our institute studies the outcome of centralization following serial corrective casting.

## MATERIALS AND METHODS

This prospective study was conducted from January 2004 to April 2008 at our institute. This study included 17 cases with 18 radial clubhands of Heikel's Grade III and IV (with average age 11 months (range 20 days – 24 months) with M:F of 2.6:1, were treated by gradual soft tissue stretching using corrective cast (14 cases) and JESS distraction (4 cases), followed by centralization (16 cases) or radialization (2 cases) and tendon transfers.

Other Heikel types were left out because no operative intervention was required in those cases. Moreover, only one case with type IV deformity with multiple congenital anomalies not compatible with life was seen and was excluded from the study. A detailed history with special emphasis on any drug or radiation exposure during pregnancy, malnutrition during pregnancy, any illness during pregnancy, similar deformities in siblings, developmental milestones attainment, and any associated illness and any treatment taken for the same was taken.

All patients were evaluated clinically. Other associated anomalies that were either evident on inspection and examination or were previously diagnosed were noted. Patients were referred to pediatrician to rule out any life-threatening associated anomaly.

The deformity was measured with the help of a goniometer. Radial deviation and volar flexion of wrist at rest was measured to find out the initial deformity. Movements at elbow and fingers were noted. The movements of fingers were taken individually of each finger as total digital motion, i.e., the sum of the movement at metacarpophalangeal joint (MCP joint), proximal interphalangeal joint (PIP joint), and distal interphalangeal joint (DIP joint). The status of thumb was noted based on the classification of Heikel^1^ [Table 2].

**Table 2:** Heikel's classification of thumb in radial clubhand

Type I	Normal thumb
Type II	Hypoplastic, deviated from normal in size, shape and position but not rudimentary
Type III	Rudimentary thumb, attached to the hand by soft tissue pedicle; passive mobility only
Type IV	Complete absence of thumb

Radiological assessment was done with full length anteroposterior and lateral views of hand, forearm, and humerus. The length of ulna and humerus, angle of ulnar bowing and, third metacarpal to ulna angle in both views were measured. The patients were also screened radiologically to find whether any evident deformities were present in other areas like chest, spine, and hip.

Hematological investigations in form of hemoglobin, total leucocyte counts, differential leucocyte counts, and platelet counts were done to rule out any associated hematological derangements.

Finally, counseling of the parents was undertaken, to explain the procedure, its complications, and prognosis, and written consent was obtained for participation in study. Rehabilitation program was discussed with the parents.

On the first arrival, if the child was less than 2 months of age, the mother was taught passive stretching of radial structures to be performed at each feeding and at bedtime. Corrective casting was delayed until the forearm was long enough to accommodate a cast.

Corrective casting was applied as advised by Flatt^2^ in three parts. The hand was first enclosed in plaster, the thumb was excluded, but the fingers were included in the early casts for good purchase. The hand was then correctly placed on the forearm, and the wrist and lower forearm were enclosed in the plaster. Finally, the cast was extended high up on the arm over the elbow, which was flexed 90°. In patients with elbow movement less than 90°, flexion was gradually increased to attain at least 90° of flexion at the elbow.

**Figure 1:**
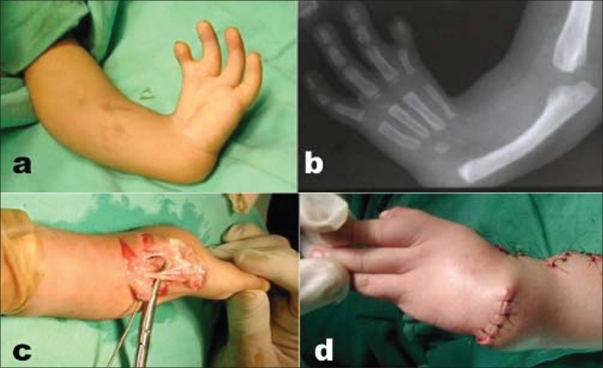
(Case no. 9) (a) clinical photograph showing grade IV radial club hand left side (b) X-ray forearm (anteroposterior view) with elbow, wrist and hand showing radial club hand grade IV (c) peroperative photograph showing the abnormal median nerve with its cutaneous branches (d) clinical photograph showing correction attained immediate post operatively

Corrective casts were changed at 2-week intervals, until there was a complete stretching of radial structures, and the hand could be passively and easily put in corrected position. This took an average of 4-6 cast change. At this stage, centralization was planned [Figure 1].

In four cases, where the deformity was very rigid, and was not yielding to spinting, JESS distraction was done to attain alignment of the hand over the forearm, prior to centralization.

### Surgical procedure

A lazy ‘S’ or ‘C’ dorsal midline incision, or a longitudinal ‘Z’ plasty on radial side with transverse incision on ulnar side, or a bilobed incision was used depending on the amount of initial correction obtained and the amount of redundant skin left in ulnar side. Once the incision is made and fascia is cut, the first structure identified is the superficial and abnormally lying median nerve with its branches on dorsoradial aspect of the wrist. The extensor tendons were identified, and a thorough dissection was done to release any tight radial structure. The extensor carpi ulnaris tendon was identified and dissected free, and the extensor digitorum tendons were retracted exposing the wrist capsule. The capsule was incised transversely exposing the distal ulna. The cartilaginous mass of carpal bones was identified. The radial anlage was divided whenever found. After proper separation of the extensors and the nerve, the ulna was freed from surroundings. A slot was cut in the proximal carpal row, with its depth equal to width of distal ulna. The cartilage of the distal ulna was shaved with a sharp blade if needed to fit into the carpal notch. A K-wire was passed through this slot into the second metacarpal and was retrogradely pushed into the ulna after seating the ulna in the notch. The size of K-wire used was as thick as to fill the diameter of the metacarpal as recommended by Goldberg.^14^ While passing the K-wire, repeated attempts to place it exactly in the medullary canal were not made, so as to prevent damage to the distal ulnar epiphysis. Therefore, not more than two attempts were made to pin the ulna. If the angulation of the ulna demanded osteotomy (i.e., >30°), then as recommended by Goldberg,^14^ the K-wire was passed into the medullary canal of the ulna and where the wire exits, which occurs at the point of maximum deformity; a closing wedge osteotomy was performed so that the K-wire now passes smoothly up to the tip of olecranon. No special calculations to calculate the CORA of deformity were made as this method was seen to give good results. The ulnar side of the wrist was stabilized by imbricating the capsule or suturing the distal capsule to the periosteum of the distal ulna. Additional stability was attained by advancing or reefing the extensor carpi ulnaris that loosens as a result of correction. The wrist was immobilized in an above elbow plaster cast in maximum elbow flexion and mid prone position.

Postoperatively, the limb was elevated and observed for any swelling, discoloration of the fingers, and stretch pain. Assisted finger movement was done by the mothers for first 48 hours. Sutures were removed on 10^th^ day, and the plaster cast was changed. The K-wire was left *in situ*. Plaster cast was changed at monthly intervals until 3 months when the cast was removed, and a below elbow polypropylene splint with radial support was applied and continued for another 6 months, after which this splint was removed in day time and used only at night. During this period of splinting, the parents were advised to passively mobilize all the finger joints and elbow and promote the child to use the hand in day-to-day activities so as to develop prehension with the corrected wrist position.

Follow-up evaluation was done at regular intervals of 3 months. The average follow-up period was 1.5 years (range 8 months to 2.5 years). At final follow-up, the range of motion of the fingers and elbow was recorded. Radiological evaluation to find change in length of ulna, change in bowing of ulna, and hand to forearm alignment with third metacarpal to ulna angle in anteroposterior (AP) and lateral views were recorded. Final data were compared with the data at their initial presentation to find out the amount of growth the ulna has attained during this period, to find out any increase in ulnar bowing, and to see if the hand forearm alignment is maintained. The final assessment of the result was made based on criteria of Bora.^15^ The results were classified satisfactory if the third metacarpal is aligned in long axis of the distal part of the ulna in AP and lateral views, no increase in ulnar bowing, and longitudinal measurement of the ulna had increased at least 50% of the projected normal increase during the interval in which patient has been followed.

The unsatisfactory result will be if any of the above criteria are not fulfilled. Results were also evaluated using the criteria of Bayne and Klug^16^ where recurrence of up to 30° was considered acceptable.

## RESULTS

Seventeen cases with 18 radial clubhands, with all congenital cases average age 11 months (range 20 days – 24 months) were treated by gradual soft tissue stretching using corrective cast (n=14) or JESS distraction (n=4), followed by centralization (n=16) or radialization (n=2) and tendon transfers [Table 3].

**Table 3:** Clinical details of patients

S. No.	Age (months)	Sex	Side involved	Side operated	Heikel's Type	Status of thumb	Associated anomalies
1	18	F	L	L	IV	Rudimentary	None
2	2	M	L	L	IV	Hypoplastic	None
3	8	M	B/L	L	IV	Hypoplastic	None
4	24	F	L	L	III	Absent	Scoliosis foot drop, short stature
5	20 days	F	L	L	IV	Rudimentary	None
6	18	F	R	R	III	Hypoplastic	Cleft Hand
7	4	M	B/L	L	IV	Absent	None
8	4	M	B/L	R	IV	Absent	None
9	12	M	L	L	IV	Absent	Tracheo oesophageal fistula, Microtia
10	12	M	L	L	III	Rudimentary	None
11	12	F	B/L	L	IV	Absent	None
12	24	M	R	R	IV	Absent	None
13	7	M	B/L	L	IV	Rudimentary	Squint
14	7	M	B/L	R	IV	Absent	None
15	4.5	M	B/L	L	IV	Hypoplastic	Torticollis
16	30	M	L	L	IV	Absent	None
17	5	M	B/L	L	IV	Absent	None
18	12	M	L	L	IV	Absent	None

The average radial deviation of wrist at presentation was 81°, while the average volar flexion was 37°. The average third metacarpal to distal ulna angle in AP and lateral views were 61° and 42°, respectively. At the last follow-up, the average radial deviation was 10° and the volar flexion was 6°. Thus, the average correction attained during the study was 71° of radial deviation and 31° of volar flexion. The average third metacarpal to distal ulna angle in AP to lateral view at final follow-up was 7° in both views. Thus, the correction obtained was in AP and lateral views were 54° and 35°, respectively. The ulnar bowing reduced from average of 25° to 12° during the follow-up [Table 4].

**Table 4:** Deformity and range of motion (preoperative and at final follow-up)

S. No.	Radial deviation (initial) (degree)	Radial deviation (final) (degree)	Volar flexion (initial) (degree)	Volar flexion (final) (degree)	ROM elbow (initial) (degree)	ROM elbow (final) (degree)	Total digital motion (initial) (degree)				Total digital motion (final) (degree)			
								
							I	M	R	L	I	M	R	L
1	70	Nil	30	Nil	120	130	120	135	135	130	130	140	140	140
2	80	15	20	20	90	90	90	210	210	210	150	210	210	210
3	50	Nil	20	Nil	100	100	230	190	220	200	220	190	220	190
4	90	20	45	5	130	140	160	165	175	185	140	140	180	200
5	90	5	45	25	90	100	90	205	210	205	100	200	190	190
6	100	40	85	40	105	105	94	198	210	210	90	135	200	200
7	110	30	45	Nil	90	90	270	225	225	225	120	200	225	225
8	60	Nil	10	Nil	100	95	210	160	195	230	200	150	200	220
9	90	Nil	45	Nil	90	60	0	50	140	150	0	50	135	135
10	90	Nil	45	Nil	100	100	270	260	270	270	260	260	270	260
11	90	Nil	30	Nil	90	90	230	210	210	210	210	200	210	210
12	100	30	45	20	90	90	220	210	170	180	210	210	150	160
13	90	Nil	20	Nil	100	110	270	225	225	225	260	220	225	225
14	50	Nil	20	Nil	90	100	210	205	225	225	200	205	220	220
15	90	45	45	10	90	90	120	210	220	210	120	200	210	210
16	45	Nil	20	Nil	100	110	140	200	150	220	130	200	150	230
17	100	Nil	90	Nil	100	100	210	225	210	205	200	220	210	205
18	80	Nil	30	Nil	90	90	160	210	230	230	140	200	210	220
Average	81	10	37	6	99	101	187	195	206	210	166	189	202	178

The length of ulna was 67% of the length of humerus in patients with radial clubhand included in our study. The range of movement at elbow showed a small increase during the follow-up period of about 1.5 years; it increased from 99° to 101°, but the range of movement at fingers showed increase in stiffness during the follow-up. The average movements of index, middle, ring, and little fingers at presentation were 187°, 195°, 206°, and 210°, respectively. These movements reduced to 166°, 189°, 202°, and 178°, respectively, thus showing a reduction of 21°, 6°, 5°, and 52° during our short follow-up The average limb length gain during follow-up was 5 cm. Postoperatively the growth of ulna was greater than 50% of the expected growth during this period in all cases, signifying that no injury occurred to the distal ulnar epiphysis during the operative intervention [Tables 5 and 6].

**Table 5:** Ulnar angle, ulnar bow, humeral length, ulnar length, relative length of ulna to humerus (initial and final)

S. No.	Humerus length (initial) (cm)	Humerus length (final) (cm)	Ulna length (initial) (cm)	Ulna length (final) (cm)	Relative length of ulna to humerus (initial) (%)	Relative length of ulna to humerus (final) (%)	Limb length (cm)	3^rd^ MC- Ulna angle in AP view (initial) (degree)	3^rd^ MC- Ulna angle in AP view (final) (degree)	3^rd^ MC- Ulna angle in lateral view (initial) (degree)
1	10.5	13	7.5	9	72	69	4	65	0	30
2	8.8	12.4	6	6.2	68	50	4	54	4	25
3	8	10.5	4.5	5	56	47	3	50	0	20
4	12	12.5	8.2	8.5	68	68	1	18	0	30
5	8.5	13.7	4.5	7.2	53	53	8	35	0	36
6	8	13	5	7.8	62	60	8	58	34	75
7	6	10.5	5	7.5	83	71	7	95	0	70
8	7.2	12	5.7	6	79	50	5	75	3	50
9	6.2	16	4.7	8.5	76	52	13	105	0	40
10	8.5	13.5	5.8	6.2	68	46	5	48	0	30
11	9.7	11.5	5.5	6.5	57	56	3	40	0	30
12	12	14.5	7.3	9	61	62	4	88	32	98
13	10	12	6.6	7	66	58	2	59	20	10
14	12	13	7.5	8	63	61	1.5	40	0	40
15	10	12	6	6.3	60	52	2	82	30	75
16	10.8	14	5.5	7.5	51	53	5	22	0	27
17	6.5	15	5	8.5	77	57	12	100	18	70
18	6.3	15.8	5	9	79	57	13	75	0	38
Average	9	13	6	7.5	67	57	5	61	7	42

**Table 6:** Treatment given

S. No.	Preoperative distraction with cast	Preoperative distraction with JESS	Operative procedure	Osteotomy and its type	Tendon transfers	Final result
1	Yes	–	Radialization	Close wedge	ECU advancement	Satisfactory
2	Yes	–	Centralization	–	ECU advancement	Satisfactory
3	Yes	–	Centralization	–	ECU advancement	Satisfactory
4	Yes	–	Centralization	–	ECU advancement	Satisfactory
5	Yes	–	Radialization	Close wedge	ECU advancement	Satisfactory
6	Yes	–	Centralization	–	ECU advancement	Satisfactory
7	Yes	–	Centralization	–	ECU advancement	Satisfactory
8	Yes	–	Centralization	–	ECU advancement	Satisfactory
9	–	Yes	Centralization	–	ECU advancemen	Satisfactory
10	Yes	–	Centralization	Close wedge	ECU advancement	Satisfactory
11	Yes	–	Centralization	Close wedge	ECU advancement	Satisfactory
12	–	Yes	Centralization	–	–	Satisfactory
13	–	Yes	Centralization	Close wedge	FCU to ECU with advancement	Satisfactory
14	Yes	–	Centralization	Close wedge	ECU advancement	Satisfactory
15	Yes	–	Centralization	–	FCU release with ECU advancement	Satisfactory
16	Yes	–	Centralization	Close wedge	ECU advancement	Satisfactory
17	–	Yes	Centralization	Close wedge	ECU advancement	Satisfactory
18	Yes	–	Centralization	–	ECU advancement	Satisfactory

Considering the criteria of Bora, satisfactory result was shown by 9 of the 18 hands, thus giving a percentage of 50%, while 16 out of 18 hands (89%) showed good or satisfactory result based on deformity criteria of Bayne and Klug [Table 7].

**Table 7:** Final results

Result	According to Bora (No. of hands)	Percentage	According to Bayne and Klug (No. of hands)	Percentage
Satisfactory	10	53	17	89
Unsatisfactory	9	47	2	11
Total	19	100	19	100

Seven hands (37%) out of 18 showed postoperative swelling, and one hand (5%) showed post operative infection. In five hands (26%), there was spontaneous extrusion or migration of the K-wire or skin irritation. One hand (5%) showed pin tract infection. There was no vascular complication in the present study.

## DISCUSSION

The treatment of congenital radial clubhand is a challenge to hand surgeon. Despite there being a school of thought, which promotes no correction of the deformity, there have been various attempts at its surgical correction^2^–^9^,^11^,^13^ since Petit^17^ first described radial clubhand in 1733.

In contrast to the series of Bora^4^ and Goldberg,^14^ where the M:F ratio was 1.5:1, the ratio of M:F in the present series was 2.6:1.

Only Heikel's^1^ Type III and IV were included in the present study; because, the incidence of radial clubhand with complete absence of radius (Heikel's Type IV) was four times (79%) more common than other radial clubhand deformities having partial absence of radius (Heikel's type III). This incidence was in comparison with series of Bayne and Klug^16^ where 67% of radial clubhands had complete absence of radius.

**Figure 2:**
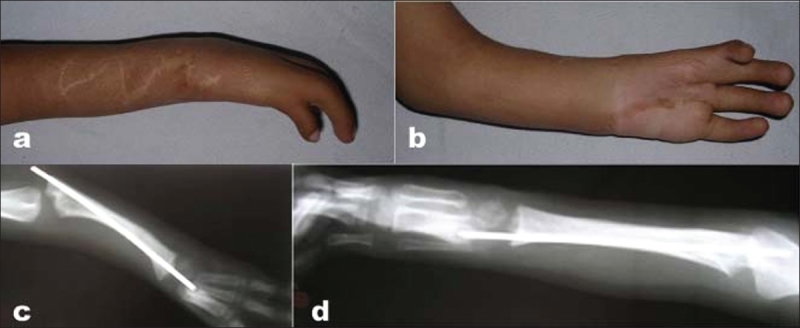
Case no 9 (a,b) clinical photograph of the same patient shown as figure 1 showing correction at 1 year follow up. (c,d) X-ray of correction of same patient at 1 year

Absence or deformity of thumb is a common association in radial clubhand. In the present series, none of the patients with true congenital defect had a normal thumb; the thumb was absent in 53%, rudimentary in 21%, and hypoplastic in 21%. In the study conducted by Pardini,^17^ the thumb was absent in 48.7%, rudimentary in 30.7%, and hypoplastic in 20.5%. Similar incidence of absence of thumb (48.5%) was reported by Bayne and Klug^16^ [Figure 1] [Figure 2].

In the present series, patients having unilateral involvement (61%) were more common than patients having bilateral involvement (39%). In the series of Pardini,^17^ 61.5% patients had unilateral involvement as compared with bilateral involvement (38.5)

In the present series, the deformity was measured by measuring the angle between the hand and the forearm by standard clinical methods. On an average, the angle between the hand and the forearm was 81° of radial deviation and 37° of volar flexion. The deformity was also quantified radiologically, by making measurements between the third metacarpal and the lower end of the ulna in both AP and lateral views. At presentation, these were found to be 61° of radial deviation and 42° of volar flexion. The difference in clinical and radiological measurements can be explained by difficulties in obtaining a standardized X-ray views and an inexact determination of lines.^1^

**Figure 3:**
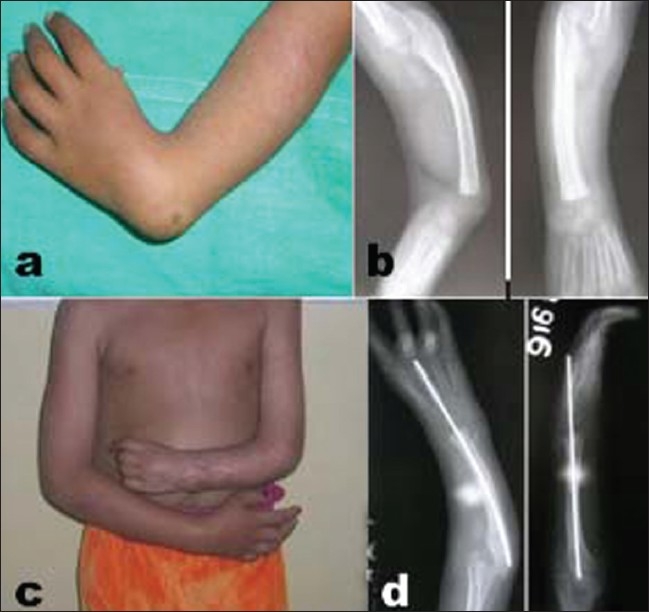
Case no 4 (a) At presentation with grade IV deformity on left side. (b) X-rays after splinting showing alignment of hand over forearm. (c) Clinical photograph at 2 years follow up after surgery showing maintenance of correction, the left forearm is short than the normal side. (d) X-rays at 2 year follow up showing retained K-wire and maintained correction

At the final follow-up, these measurements were repeated to assess the improvement attained. It was seen that clinically the average radial deviation was 10°, while the volar flexion was 6°, thus showing an average improvement of 71° of radial deviation and 36° of volar flexion. Radiologically these measurements in AP and lateral views were 7° of radial deviation and 7° of volar flexion, showing an average correction of 54° of radial deviation and 35° of volar flexion. In the 10-year follow-up study of Bora,^4^,^15^ the average hand-forearm angle in the patients treated by centralization and tendon transfer was 35°. Thus, the centralization of the carpus over the ulna proved satisfactory in correcting the deformity [Figure 3].

Stiffness of the elbow in extension is a frequent association with radial clubhand. The presence of a stiff elbow in extension is an important consideration. If the stiffness cannot be corrected, it is a definitive contraindication to any surgical procedure.^18^ In our series, two patients could not be operated on one side due to lack of adequate elbow flexion. The average range of motion in children who underwent operative intervention was 99°, with 53% (n = 10) showing elbow movement >90° and 47% (n = 9) showing elbow movement <90°. At final follow-up, there was not much change and the average elbow movement increased to 101°, with 63% (n = 12) showing movement >90° and 37% (n = 7) showing movement <90°. In the study of Heikel^1^ the average elbow motion was 60°, while it was 70° in the series of Sherik.^19^ One obvious reason for increased average movement of the elbow in our series, as compared with others, is that we measured passive elbow movement.

In our series, since we decided to leave the K-wire indefinitely inside, we did not measure the range of movement at the wrist. The amount of range of motion of fingers tended to increase progressively from index to little finger. This finding is consistent with Lamb *et al.*^12^ The average total digital motion at presentation of index finger was 187°, middle finger was 195°, ring finger was 206°, and that of little finger was 230°. These movements showed significant decline in the final follow-up where the average total digital motion of index finger was 166°, middle finger was 198°, ring finger was 201°, and that of little finger was 178°. In the study by Lamb *et al*,^13^ the average range of flexion in each digit before and after operation was as follows: index 98° and 88°, middle 101° and 102°, ring 185° and 155°, and little finger 210° and 212°. These findings of reduced motion in fingers are consistent with our findings. The average ranges in our study are more as compared with Lamb's study, because we measured passive motion, as measuring digital motion actively was not feasible in these young children. We think that there is a need to reassess these movements for a longer follow-up period, as our present follow-up is short, and the range will surely increase once the child starts using the corrected extremity to activities of daily living.

To determine the growth pattern of the forearm in this condition, the length of ulna relative to the humerus was measured. Shortening of the ulna was measured by taking the ratio of length of the humerus to the length of the ulna radiologically. In normal subjects, this ratio is 1:1. It was seen that in majority of subjects with radial clubhand, the ulna was 60-70% shorter then humerus, the average being 67%. This shortening was seen to get somewhat reduced to 57% after operative intervention, thus showing that the growth potential of the ulna was not reduced after operative intervention. According to Bora,^4^,^15^ the ulnar length in radial clubhand at birth is approximately 60% of the normal, and this discrepancy is maintained during growth.

Increase in limb length was measured by comparing the radiological lengths of humerus and ulna at presentation, minus these lengths at final follow-up. In the present study, there was an increase of average 5 cm of length of limb, during the follow-up period, after centralization as compared with the length at presentation. This finding is fallacious as the period of follow-up was neither uniform nor sufficiently long, and age of patients was variable. However, these findings help us to say that treatment by centralization or radialization of the carpus over the ulna did not produce any detrimental effect on the growth of lower ulnar epiphysis, until the last follow-up.

The bowing of the ulna is a significant problem in this deformity. It not only shortens the already short limb, but also significantly affects the appearance, despite correction of wrist position. In the present series, the average ulnar bowing was 25° in AP view. This bowing showed decrease to 12° at final follow-up. Two cases showed no bowing both at presentation and at follow-up. About 21% (n = 4) patients showed increase in ulnar bowing despite operative intervention. In one case, the bowing occurred despite the K-wire being inside leading to the bending of the K-wire. In those cases who were operated near one year of age and in those in whom ulnar osteotomy was done, bowing did not occur significantly. These patients were operated at less than 6 months of age, and osteotomy was not done. In 3 of these 4 cases, the K-wire got extruded as it was not in proper position intramedullary. In one case, the bowing occurred despite the K-wire being inside, leading to the bending of the K-wire. In those cases which were operated near one year of age, and in those in whom ulnar osteotomy was done, bowing did not occur significantly. Thus, in our opinion, operative intervention should be done at about the age of one year. In our series, ulnar osteotomy was done in 42% (n = 8) of the patients, who showed ulnar bowing >30° at the time of operative intervention. In one case, at the time of primary operation, osteotomy was not done (bowing of 0°), but during the follow-up period, the bowing was seen to increase significantly (45°), so a repeat procedure was needed to do an osteotomy and refix it with a K-wire.

In our series, tendon transfers were done in 89% (n = 17) cases. In 15 cases, ECU was either reefed or advanced. In two cases, along with advancement of ECU, FCU was attached to ECU.

The results at the final follow-up were graded on the basis of the criteria of F.W. Bora,^15^ 53% (n = 10) of the patients showed satisfactory results, whereas the results of remaining 47% (n = 9) were unsatisfactory. The results were also graded on deformity criteria of Bayne and Klug,^16^ 89% (n = 17) showed good or satisfactory result, and 11% (n = 2) showed unsatisfactory results.

In the present series, one patient (5%) had developed pressure sore in the cast, three (16%) patients developed dermatitis under the cast, two (10.5%) cases of bilobed flap incision showed marginal necrosis of the flaps, but none needed any secondary intervention for the same. Postoperative infection was seen in one case (5%). Self K-wire extrusion or migration was seen in five cases (26%). This is a significant problem as quoted by Flatt^2^ that pinning the ulna and the third metacarpal is not always easy. In two cases, repeat fixation of wrist was done after extracting the previous K-wire and letting the wound settle as the extruded implant started hurting the skin. In one case where ulna overgrew the K-wire, a repeat operation was done to remove the K-wire from the bone.

Recurrence is a problem of utmost concern in any correction of deformity. We took the overall appearance of the limb into consideration and measured the total angulation (radial deviation + ulnar bow) at the final follow-up. Total angulation of greater than 30° was considered significant as after this the deformity becomes visibly apparent. Ten patients (53%) in our series showed recurrence of deformity with mean total angle of 37° (range 18°–70°). Six patients (31%) of these 10 had significant total angulation of more than 30°. This can be compared with the results reported by Damore *et al*,^20^ who found that at end of 6.5-year follow-up in (19 clubhand) total angulation was 63° (range 20° to 120°), and the correction loss was 38°. In our series, this has been 37°. It is worth mentioning at this point that the final evaluation of correction of the deformity is not by cumbersome calculations on a radiograph, but by correction that is functionally and cosmetically acceptable.

We conclude that the centralization of carpus over the ulna with tendon transfer has proved satisfactory in correcting the deformity and producing wrist stability. There was no detrimental effect on the growth of the distal ulnar epiphysis; the range of movement at elbow showed no significant increase, while the digital motions decreased postoperatively. The findings of this study need further follow-up, as a short follow-up for evaluating such congenital deformities is not appropriate [Table 3].
